# Understanding factors affecting breastfeeding practices in one city in the Kingdom of Saudi Arabia: an interpretative phenomenological study

**DOI:** 10.1186/s13006-020-00350-4

**Published:** 2021-01-06

**Authors:** Amal Murad, Mary J. Renfrew, Andrew Symon, Heather Whitford

**Affiliations:** 1 Maternity and Childhood Department, College of Nursing, Taibah University, Al-Madinah, Saudi Arabia; 2 Mother and Infant Research Unit (MIRU), School of Health Sciences, University of Dundee, Dundee, Scotland, UK

**Keywords:** Breastfeeding, Support, Mothers, Decision, Kingdom of Saudi Arabia, Phenomenology, Lived experiences

## Abstract

**Background:**

Breastfeeding duration has declined in the Kingdom of Saudi Arabia (KSA) in recent decades, although accurate national data about different breastfeeding indicators by infant age are lacking. This qualitative study, the first in KSA, aimed to understand the factors affecting mothers’ decisions and experiences regarding any breastfeeding practices.

**Methods:**

A qualitative phenomenological approach was used to investigate mothers’ experiences of breastfeeding. Non-probability convenience sampling and snowballing strategies were designed to recruit participants. Semi-structured interviews were conducted with 16 mothers, from two hospitals and three primary health clinics in Al-Madinah city, from December 2017 to March 2018. Interpretative phenomenological analysis was the analysis framework.

**Results:**

Three themes were identified: 1) ‘Up against the system’: policies, staff and systems were the main barriers to exclusive breastfeeding; 2) ‘Social support and negativity’: family support in the first 40 postpartum days protected breastfeeding continuation and was highly appreciated, but negative comments limited breastfeeding practices thereafter; and 3) ‘Managing tensions’: mothers’ religious beliefs about breastfeeding boosted their decisions; however, the challenge of managing tensions influenced mothers to stop breastfeeding earlier than they wished. The study revealed that mothers had no doubts about wanting to breastfeed their babies; but continuation was adversely affected by unhelpful hospital policies and staff actions, the lack of ongoing social support, and by others people’s negativity, rather than by the mothers’ own views. Stopping breastfeeding earlier than planned was a complex decision for most mothers. However, mothers said that they intended to breastfeed their next baby successfully.

**Conclusions:**

Healthcare professionals (maternity staff, paediatricians and pharmacists) need education and training to support exclusive breastfeeding effectively. Increasing the number of hospitals with Baby Friendly Hospital Initiative accreditation, which includes staff practice changes, is needed to protect and support exclusive breastfeeding. Ongoing professional and peer support, and improving conditions at workplaces and universities, are needed to help mothers to continue breastfeeding successfully. Effective, coordinated national policies can support mothers’ decisions in relation to breastfeeding. Such changes will reduce the tensions experienced by women and help them to achieve their breastfeeding goals and to breastfeed for longer.

## Background

The Kingdom of Saudi Arabia (KSA) has witnessed noticeable declines in breastfeeding over the last few decades. Thirty years ago, a national survey conducted in most Saudi regions found that the rate of any breastfeeding at 2 years of age was 62% [1]. However, the most recent national survey, conducted in 2004–2005, found that the rate of any breastfeeding at 12 months was only 1.8% [2]. The rate of exclusive breastfeeding by age has not been reported; and available data on other breastfeeding practices uses differing definitions making comparisons and trends difficult to ascertain [3]. There is also a lack of planning to support effective evidence-based actions [3, 4], and in KSA only 26 hospitals out of 415 (6.2%) were designated or reassessed as a Baby Friendly Hospital Initiative (BFHI) between 2010 and 2015 [4]. All cross-sectional surveys/questionnaires conducted to date have concluded that the current practice of breastfeeding is far from compliant with the WHO recommendations [2, 3]. Moreover, the mother’s perception of having insufficient breast milk has been the most commonly reported reason for early breastfeeding cessation [5, 6], indicating a lack of professional support. Other factors include lack of support for women who return to work or education while still breastfeeding [7] and maternal age, as younger mothers introduced prepared food earlier than older mothers, most from the third month [8]. However, all these reports [2, 3, 5–8] are from surveys of variable quality and there have been no qualitative studies of the experiences of breastfeeding women in KSA.

Therefore, this study was planned to investigate and understand mothers’ lived experiences with breastfeeding, and to explore the factors influencing their decisions.

## Methods

### Research site and sampling

This study, using interpretative phenomenological analysis [9], was carried out in two government hospitals and three primary healthcare centres located in different regions of Al-Madinah city in KSA. This allowed recruitment of mothers with a diverse range of socio-demographic characteristics and breastfeeding experiences.

Non-probability convenience sampling and snowballing strategies were used to recruit participants. Mothers who had a baby under 2 years old, had breastfed their baby (regardless of the frequency and the duration), and who understood and spoke Arabic were eligible, while mothers who had babies older than 2 years or did not speak or understand Arabic were excluded.

### Recruitment and interviews

All recruitment and interviews were conducted in Arabic by Amal Murad (AM). Posters providing basic study information and a contact phone number were displayed and flyers distributed in the five healthcare settings, on WhatsApp, and sent to friends and colleagues.

When potential participants made contact, they were sent a participant information sheet. A place and time for the interview was held in a quiet room: either in the healthcare facility, nursing college or a community centre. Consent was sought at the start of the interview.

A pilot study with two mothers was carried out before the main data collection to develop and test the questionnaire but these data were not included in the analysis. Sixteen women participated in the main study, with interviews conducted within a three-month period. Open questions, with follow up probes, were derived from previous studies and asked about mothers’ lived experiences concerning breastfeeding.

Interviews were audio-recorded and transcribed afterwards to capture the mothers’ words, expressed feelings, and spoken dialect. The researcher, who speaks English fluently, translated all the transcripts from Arabic into English, ensuring that the original Arabic meaning was conserved in order to maintain the mother’s ‘lived experiences’ [9].

To ensure the cross-cultural research translation process had captured “conceptual equivalence” [10], and maintained rigour [11], three English transcripts were independently back translated into Arabic by an expert academic whose first language was Arabic. Any differences or disagreements over translation were identified and discussed to achieve the most accurate culturally equivalent meaning [12]. The final equivalent English transcripts were exported into the software NVIVO 11 for managing the data analysis process.

### Process of interpretative phenomenological analysis

Interpretative phenomenological analysis allows in-depth and detailed investigation into the participants’ experience [9]. It also is a participant-centred approach [13], with the meaning of the participant’s lived experience being understood by the researcher through an interpretative process [9]. The key quality control strategy is reflexivity. Thus the researcher’s characteristics, such as gender, language, being a mother, health professional and living in the same city as the participants, were considered by AM when conducting and analysing the interviews.

Analysis involved extensive reading and re-reading, and exploration of different descriptive, linguistic and conceptual comments [9]. Similarities, connections and relationships were identified and sub-themes created. The final step was to look for similarities and patterns across the entire group of sub-themes to form main themes.

The robustness and validation of the analysis process were assured by reflexivity, confirmation by co-authors (Andrew Symon and Heather Whitford), and discussions on the process and development of themes continued throughout the analysis with all co-authors. There were major agreements and similarities regarding the developed themes; with most discussion about refining the names of the themes.

## Results

### Socio-demographic characteristics

Similar to the overall educational level of women in Saudi [14], the majority of the participants were educated to degree level (Table 1). The majority of participants were either working or studying, and infants’ ages ranged from 2 months to 2 years at time of interview.

**Table 1:** Socio-demographic characteristics of participants

	Numbers
**Age (years)**	
20–24	2
25–29	6
30–34	5
35–39	2
40–44	1
**Educational level**	
Secondary school	6
Degree	7
Postgraduate	3
**Employment status**	
Housewife	7
Student	3
Employed	6
**Age of last child (months)**	
Under 6	4
6–12	5
Over 12	7

Three main themes emerged: 1) ‘Up against the system’; 2) ‘Social support and negativity’; and 3) ‘Managing tensions’ (Table 2). Each main theme consisted of sub-themes and each sub-theme consisted of a breastfeeding facilitator and several barriers to breastfeeding. All these themes were developed from the participants’ reports during the analysis process. Pseudonyms are used throughout.

**Table 2:** The main themes and sub-themes

Main Themes	Sub-themes	
	Facilitators	Barriers
**Up against the system**	Breastfeeding information and advice	Do it yourself Breastfeeding discouragement Unhelpful advice Unfriendly environment
**Social support and negativity**	Breastfeeding encouragement and support	Negative comments Conflicting advice Poor understanding of breastfeeding needs
**Managing tension**	Belief	Multiple roles Concerns and worries

#### Up against the system

This theme was the principal reported factor that affected mothers’ decisions, particularly about exclusive breastfeeding. Healthcare professionals were reported as consistently advising mothers to breastfeed. However, unhelpful staff, discouraging policies and working/studying environments that inhibited breastfeeding meant that mothers felt they were ‘up against the system’ when trying to breastfeed exclusively.

Mothers reported that breastfeeding information in healthcare settings was a vital facilitator, and that receiving specific advice and support was their principal source of encouragement.

*Dalia: I had breastfeeding leaflets from the hospital for my first baby till the last one from the clinics and wards. I read . . . read all breastfeeding papers . . . handouts . . . mm . . . they gave me [information] each time I give birth, that’s why I know it’s [breastfeeding] important.*

Participants reported being advised directly by staff to breastfeed during pregnancy, and/or once they gave birth, and/or before leaving hospital. This also gave them encouragement to breastfeed when the staff put the babies on their mothers’ chests or handed them to the mothers:

*Besma: She [a nurse] talked to me after delivery. She advised me to let my baby suck my breast very well to get used to sucking my breast from early days.*

However, the majority reported that policy, staff and systems were crucial barriers to continuing to breastfeed. Participants felt they were left to ‘do it yourself’ once they had started. Most mothers reported that they had been left unaided when breastfeeding, without any effective support:

*Ola: the first time for breastfeeding was on the second day after birth [because the staff kept her baby in the nursery due to the hospital policy]. I went there again [nursery] and held my baby and put her on my breast, but I had no previous experience with natural feeding . . . I was left alone with my baby in a corner . . .*

“*Natural feeding”* is an Arabic phrase that was reported by mothers and it means *breastfeeding* in English. Mothers also commonly reported being discouraged from breastfeeding, for example because of their health status – such as having had a caesarean section (CS):

*Gala: I requested my baby but they refused and told me “you are tired and exhausted, it’s contraindicated”. I also asked them to bring my baby to be with me in the same room because I wanted to look at him to stimulate my breast for milk production, they also refused that. I also asked them to bring my baby when he wanted a feed because I wanted to feed him from my breast, but they said “you cannot breastfeed him because you are under analgesia and anaesthesia drugs”.*

Unhelpful advice was reported by the majority of mothers if, after a few weeks or months, they sought guidance from paediatricians or pharmacists. These professionals encouraged and/or instructed the mothers to combine breastfeeding with infant formula.

*Fulla: I told the paediatrician that “my baby is not sucking my breast very well . . . he told me “your baby is very hungry he’s crying because he’s hungry”. He told me to feed him artificial food if he didn’t take or refused my breasts.*

Most of the mothers who were studying or who had returned to work reported an unfriendly environment. They reported the inconvenience of trying to combine breastfeeding with full-time working or studying and that there were no breastfeeding facilities. For example, when Isra explained that she needed to leave work early to continue breastfeeding, staff refused her request.

These mothers reported that they suffered from moderate to severe breast engorgement. However, they managed to persevere with breastfeeding.

Students reported that the university environment was unsupportive and unfriendly towards breastfeeding mothers and that they had to attend all lectures and do the same exams and amount of homework as students without children. Mothers at university or at work reported that they used bottles for expressed breast milk and/or sometimes combined breastfeeding with formula.

*Kady: He was three months old when I returned to college . . . at the beginning I faced issues because my breast was engorged and became like a rock because I’m not feeding my baby for hours and I didn’t know where to breast pump at the university . . . and as I told you, I was patient until I finished my lectures, then I went back home.*

#### Social support and negativity

Support was the main facilitator that enabled mothers to continue breastfeeding their babies after hospital discharge, but people’s negative comments limited women’s ability to continue breastfeeding.

The mothers described the comprehensive and different types of support - practical, psychological, financial and nutritional - that they received from a variety of close people, particularly the baby’s maternal grandmother, during the first 40 days. All of the mothers reported that this Arabic cultural care norm was known either as *“the 40 days”* or “*Nufas/Al-Nufas days*” (Nufas means postpartum). Overwhelmingly, mothers reported being highly appreciative of postpartum support.

The principal encouragement from family members during this time was to feed colostrum, to continue breastfeeding, as well as being helped with breastfeeding positioning and being provided with different types of breastfeeding support.

*Ola: At mum’s house, my sisters were there and taught me the steps of breastfeeding . . . the holding, cuddling and positioning . . .*

Husbands were also reported as having a crucial role to play:

*Nuha: My husband encouraged me to breastfeed my baby everywhere —at land, air and sea. He told me not to be embarrassed in public places and if I felt embarrassed, he would stand in front of me if I wanted. He asked me to continue breastfeeding [not in a hurry] several times.*

Nonetheless, negativity was a critical barrier to breastfeeding; negative comments and criticism of both mothers and babies were reported. The most common unsolicited Arabic word from other women was *“Ya-haram”*. There is no direct English equivalent but, essentially, it means “oh, poor baby” or “oh, how sad” or “oh, insufficient milk”. Mothers declared this the most disliked word:

*Kady: “Oh, Ya-haram, you have insufficient milk, or your baby needs more milk and you don’t have enough. Oh, your baby is still hungry.”*

*Meha: my grandmothers and his grandmothers kept repeating this phrase: “You have no milk, your breast has no milk, give him a bottle.” All people around me were repeating this expression: “Ya-haram you have no breast milk.”*

Other distressing comments referred to mothers’ breast becoming saggy, or their body size and shape being imperfect. These comments influenced the mothers’ feelings since they reported that they were being scrutinised both visually and verbally when nurturing their babies in front of relatives and friends.

Conflicting advice was also commonly reported when the mothers faced breastfeeding challenges, such as family members not understanding why breastfeeding was challenging.

*Fulla: Mum told me: "Why are you doing that? [following the steps of breast attachment as she learned from educators at hospital] Just pick up your baby and put him on your breast . . . you don't know how to breastfeed your baby!"*

Poor understanding by others of breastfeeding needs arose, particularly when these changed. This often occurred after mothers returned to university or work, because they were working or studying full-time.

Fulla, like several mothers, stated that her husband initially supported her breastfeeding decisions, but then started arguing about adding artificial milk:

*Fulla: “If it’s as bad as what you are saying, they wouldn’t sell it in pharmacies, they wouldn’t use it in hospitals, they wouldn’t sell it with medications that treat people and kids.” So, after this argument I just give him [baby] a bottle of artificial milk.*

#### Managing tension

Mothers believed in breastfeeding and they highlighted their willingness to achieve their breastfeeding goal with their next child, but the tension that resulted from multiple roles and concerns led them to stop breastfeeding earlier.

Belief in breastfeeding as the natural source of baby milk supported mothers’ decisions. This often related to distinguishing *“natural feeding”* and *“artificial feeding/milk”.* “*Natural feeding”, “the feeding”, “feeding them naturally”, “feeding them natural milk”* were reported by mothers and they all mean *breastfeeding* in English. *“Artificial feeding” and “Artificial milk”* mean formula; all were also reported by mothers.

*Besma: I decided to breastfeed … (and) to continue breastfeeding because I want to feed my baby naturally. I feel and believe that natural feeding . . . causes less suffering with the newborn baby.*

*Esmat: only the natural milk [breastfeeding] has full benefits for babies.*

Emotional communication between mothers and babies during breastfeeding was the main reported self-belief that encouraged mothers to continue breastfeeding. Several added that they felt more kindness and tenderness when nursing their babies.

*Lena: I love feeding him naturally and I’m feeding him my love*

Overwhelmingly, they believed in breastfeeding because it was recommended by Allah; this was the foremost reported reason for insisting on or persisting with breastfeeding. Several mothers cited the Quran in support of this.

*Asma: Two years definitely, I’ll breastfeed him for two years as Allah says in the Quran: “mothers, who gave birth, shall breastfeed their babies two complete years” . . . there is wholesomeness from this verse; it’s the Quranic foresight.*

The following mother clarified her belief in the Quranic instructions that extended to the responsibilities of the father to his wife during breastfeeding:

*Nuha: There are other things (verses) and I was stressing the idea of nursing expenditure (laughter). For me as a nursing mother, I need to feel well, so presents and such stuff make me feel good and I took advantage of these religious recommendations (laughter).*

However, most mothers faced challenges after a few months of juggling multiple roles, leading some to stop breastfeeding earlier than they had wished. This was particularly so for mothers who were working or studying, or who had health issues.

*Ola: I gave her natural milk and artificial milk, both. I only used the artificial when I was busy. For example, when I had to go for follow-up appointments or when I was in a lecture and she was in nursery . . . and it’s out of my hands [forced factors].*

Concerns were commonly reported when mothers worried about their babies’ crying, sleeping and preferring formula:

*Nuha: So she [infant] started to love artificial milk before I returned to work and I felt that she accepted artificial milk more than my breast milk, so I knew that she liked to take milk from the bottle, not from my breasts.*

Some mothers limited breastfeeding in public and sometimes covered their breast because they either believed or had been advised not to practise frequent and long breastfeeding in front of other women in order to avoid *“the eye” or “evil eye”. “The eye”* and *“evil eye”* were concepts believed by several mothers in this study; it affected their breastfeeding practises in front of other women.

*Fulla: a bad thing would happen to you as result of evil eyes . . . for example, I wanted to breastfeed my baby in front of other women but sometimes my relatives prevented me because they said “be careful of evil eyes!!”*

Most mothers said that they breastfed in front of children and other women but only in front of close male relatives. However, they made specific arrangements for their personal beliefs when they breastfed in the presence of male non-relatives in public places by covering their breast with a light scarf or fabric, adjusting their seating, or trying to find a private women’s section.

*Esmat: I breastfeed everywhere and every time and just cover the upper side [pointed on her shoulder] in front of non-relative men*

## Discussion

This qualitative research is the first study in KSA in which mothers’ voices were listened to in regard to their breastfeeding experiences and decisions. Choosing interpretative phenomenological analysis facilitated richer and deeper data on factors affecting women’s experiences. However, data from only 16 mothers cannot be widely generalised.

Our research identified three main themes affecting mothers’ decisions and experiences regarding breastfeeding; ‘up against the system’, ‘social support and negativity’ and ‘managing tensions’.

We found that breastfeeding information from healthcare professionals in KSA predominantly encouraged mothers to breastfeed. Similarly, a recent survey in KSA found that mothers who received breastfeeding information during their pregnancy were significantly more likely to initiate breastfeeding early (≤1 h) [15].

However, policies, staff and systems were the main barriers to the initiation of and to exclusive breastfeeding. Similarly, surveys in both Jordan [16] and Lebanon [17] have identified that hospital policies and staff actions were barriers to breastfeeding initiation. These barriers also affected breastfeeding continuation during the first month in Lebanon [18].

Mothers reported unhelpful advice about breastfeeding and being encouraged to formula feed. This echoes a US study by Taveras et al [19] which found that mothers were more likely to stop EBF by the fourth month, when paediatricians recommended that they added formula feeding. There is evidence that staff in healthcare settings need evidence-based education and training to effectively support women to breastfeed [20, 21]. The Baby Friendly Hospital Initiative (BFHI) has been shown to improve professionals’ breastfeeding knowledge and attitudes [22]; and had a successful impact on breastfeeding initiation, duration and exclusivity [23].

We found that unfriendly environments at work and in universities were also critical breastfeeding barriers. Other cross-sectional studies in the Middle East have found that working mothers were more likely to stop breastfeeding before 6 months: in the KSA [24], Kuwait [25], Oman [26] and Lebanon [27]. The absence of specific measures to protect breastfeeding in the workplace has been noted to lower breastfeeding duration in both the Middle East [28] and the USA [29]. Implementing adequate protective breastfeeding policies in workplaces, such as paid maternity leave and a breastfeeding space have been found to improve breastfeeding outcomes [20, 21].

We found that social and familial support, particularly in the first 40 days postpartum, was a very important facilitator in terms of continuing breastfeeding. Similar findings have been reported in Iraqi women in Australia [30] and Lebanon [31]. Quantitative studies in Iraq [32] and Kuwait [33] found that grandmothers assist breastfeeding by encouraging colostrum feeding, breastfeeding on demand, and by helping with childcare. However, our study revealed that several mothers were receiving conflicting advice such as discouraging formula but encouraging herbal drinks. Emirati grandmothers, while supporting breastfeeding and discouraging artificial feeding, have also been reported to encourage adding traditional fluids during the first 6 months [34].

We found that negative comments notably limited breastfeeding. Although the literature does not clearly explore people’s comments about mothers’ breasts from Middle-Eastern countries, studies in Australia [35] and in the UK [36] have acknowledged that judging a woman’s body can discourage mothers from continuing to breastfeed in public. Though the UK and Australia are different societies, it can be argued that worldwide, women’s breastfeeding practices are commonly affected by others’ comments.

A strong belief in the importance of breastfeeding was an empowering factor, a finding which has also been noted in Beirut [31], Syria and Jordan [37] and in a multi-ethnic study in the UK [38]. Our findings revealed that some of this empowerment derived from religious counsel. Islamic perspectives on breastfeeding have also been reported to motivate non-Arabic Muslim mothers and to encourage positive attitudes to breastfeeding in Malaysia [39], the USA [40], Bosnia [41], Australia [42] and the UK [43].

Mothers who were juggling multiple roles and who had other concerns about their infant’s feeding were more likely to stop EBF before 6 months and to stop breastfeeding earlier than planned. This echoed the findings of Nabulsi’s [31] Lebanese study which found that sleep deprivation, exhaustion, maternal employment and fear of having ‘bad milk’ due to stress were commonly identified reasons for stopping after a few months.

Our study’s novel findings include concerns that ‘the eye’ or ‘evil eye’ disturbed mothers and interrupted breastfeeding. The evil eye concept has been reported in Turkey [44], in relation to the belief that breast milk may become unclean and infected, and in KSA in relation to illness [45, 46]. Our study is the first in the region which has found that breastfeeding was interrupted because of maternal worries regarding breastfeeding in front of other women because of ‘the eye’ or ‘evil eye’.

### Strengths and limitations

The quality of this research was achieved by the coherence of the philosophical methodology, data transparency by presenting the participants’ extracts, and thoroughness of analysis. The sample was small and the participants in this study did not represent a wide range of socio-demographic characteristics so the data cannot be widely generalised. However, as the first qualitative study of breastfeeding experiences conducted in KSA, the findings present consistent views suggesting that their experiences were widely shared and provide a foundation for future studies.

## Conclusions and recommendations

This study found that in spite of the commitment of mothers and cultural support from families, breastfeeding in KSA is challenging. Declining rates could be attributed to inaccurate and unhelpful advice from healthcare professionals and a lack of workplace policies. Furthermore, working and studying mothers did not have the practical support in the workplace and universities to continue to breastfeed. To reverse this decline, evidence-based education and training for healthcare professionals are needed for successful EBF. Improving workplace and university conditions and policies are also needed to improve breastfeeding duration and protect women’s breastfeeding rights.

Having evidence-based, formal, consistent and helpful breastfeeding support can also increase the EBF rate and duration. More effective collaboration between national systems for breastfeeding support is needed to resolve challenges in education and workplaces. The collaboration can be achieved by recognising the importance of implementing BFHI. The valuable cultural tradition of family support for 40 days should be promoted professionally. Enhancing mothers’ ability to manage tensions will help them to achieve their breastfeeding goals. Thus, programmes such as the BFHI could improve institutional practices, community support and breastfeeding outcome [47]. There is also an urgent need for utilising routine breastfeeding data effectively, including improving breastfeeding indicators to produce reliable data [48]. Future research is needed, to include both in-depth and representative studies.

## Data Availability

The dataset (transcripts and consents) is available from the corresponding authors on reasonable request.

## Abbreviations

BFHI: Baby Friendly Hospital Initiative
EBF: Exclusive breastfeeding
IPA: Interpretative phenomenological analysis
KSA: Kingdom of Saudi Arabia
SNHS: School of Nursing and Health Sciences
UAE: United Arab Emirates
